# Essays and Addresses

**Published:** 1898-03

**Authors:** 


**Essays and Addresses.** By Sir J. RUSSELL REYNOLDS, Bart., F.R.S., M.D. Pp. xxiv., 307. London: Macmillan and Co. Limited. 1896.

These Essays and Addresses, as might be expected of so distinguished an author, have a very wide range, and are directed in great measure to first principles. As a teacher and examiner he had his own views on the course of medical studies, and he considered that far too much time is spent by the average student over anatomy. He complains that positivism is permeating all our scientific work and directing the course of our medical enquiries, and that physiology is taking a second place; and he draws a painful picture of the struggling student and practitioner, to whom certain facts have been taught instead of principles.

The essays are permeated with the idea of "Life" as a central object of study: life, as the aim of the physician, that which gives unity and form to all his actions, purposes, and thoughts. A patient is not a clinical phenomenon, but a being holding an individual history: and all examination of a patient should be based on philosophical methods. This ideal naturally leads to a dislike of specifics in treatment, and also to disapprobation of that mental method in some doctors, who are content in recognising a definite lesion in a patient, and not to treat him as a whole. In some patients there may be nothing organically wrong to be discovered; but there may be in others the consciousness of a deep unrest, or of a failing power, which he feels, but he cannot see, or of a sense of impending evil in brain or heart, or of a want of intellectual grasp. In fact, the author does not believe that precise observations should be limited only to a class of facts, which can be counted, measured, or weighed. Specialism may be abused; but the adequate equipment of the country practitioner, that makes him ready for any kind of emergency, and absolutely free from the vice of specialism, has depended originally, more or less, on the work of specialists.

As Harveian orator, Sir Russell Reynolds's chief idea seems to have been, that in his great work Harvey reaped much that he had not sown, but he also sowed much more which others after him might reap. "The thoughts of men are widened by the progress of the suns."

In the address the author of these essays prepared for the Bristol Medical Students, he spoke of the value of competition. He considered that final examinations were faced with far greater ease, if the student had competed intellectually every year with others in local struggles.

Perhaps one of the most striking of these papers is his address on sanitary science and preventive medicine, in which he goes much farther back than is usually done in these studies, and discusses the causes of disease which are inherent in the individual and those which are brought to bear upon him from the outside.

We leave untouched the interesting account of some of the early teachers in University College, as also the essay which commends to students the highest principles of religion; but Reynolds held in high regard his teachers and colleagues, and his wise outspoken words to students teem with the deepest Christianity.

The volume fitly closes with the Presidential Address given in London, not long before the author's death, at the annual meeting of the British Medical Association. The keynote was the power of living things for good or evil; viz., in the conservation of health, and in the prevention and cure of disease. In many respects this address was the outcome of his professional thought during his whole life. Accurate as he was in all medical work, his freshness and his power were founded on the wide views he took of life, his dislike to be limited to this or that theory, to this or that line of practice, his possession of "an incisive scientific perception, associated with a profound sense of those hidden laws of life, which evade analysis, but cannot be disregarded by genuinely intellectual minds": and these essays are a picture of the man.

